# Supplementary material for: Costing the intergovernmental negotiating body (INB) process
Source: PLOS Glob Public Health. 2024 Dec 11;4(12):e0003978. doi: 10.1371/journal.pgph.0003978 (PMC11633964; doi:10.1371/journal.pgph.0003978)
Supplement: Attachment — Submitted filename: response to reviewers PlosGPH costing INB.docx [file pgph.0003978.s001.docx]

Reviewer #1: Thank you for the opportunity to review this manuscript - which I genuinely enjoyed reading.
What a fabulous paper and wonderfully creative research area, which offers a different perspective on INB proceedings. I greatly enjoyed reading this paper. There is enough logic in the calculations to support its publication, as one of sufficient methodological-rigor.
Accept.

Thank you so much for this encouraging review, we appreciate that you think there is merit in the research idea, and indeed methodology.

Reviewer #2: Peer Review Report for Costing the INB Process

Thank you for the opportunity to review this manuscript. I’ve taken the time to carefully go through the paper and supporting documents, and I’m providing feedback to help strengthen the analysis and maximize its impact.

Thank you so much for this detailed and thoughtful feedback on our paper – we are very appreciative of it, and believe the paper is stronger now as a consequence.

Overall, I like this paper. It’s timely and relevant, offering an important analysis of the financial costs tied to the Intergovernmental Negotiating Body (INB) process. Quantifying these costs is an important step toward understanding how resources are allocated within global governance—particularly in the context of global health. The analysis is especially valuable given the growing conversations about the purpose and utility of international legal agreements, pandemic preparedness, governance structures, and the WHO’s role in all this. The paper stands out by drawing attention to the opportunity costs of these negotiations compared to more direct investments in health security.

We are pleased that you think there is merit in the paper’s idea, aims and indeed analysis.

However, there are several areas where further clarification and development would enhance the robustness and clarity of the work. I offer the following suggestions:

1. While the paper clearly sets out to quantify the costs of negotiating the pandemic agreement, the introduction could do more to position this research within the literature. The authors mention the lack of prior methodologies, but it would help to explicitly state that this is among the first studies to provide a detailed financial estimate of treaty-making in global health. Also, consider a statement on why this matters—perhaps asking whether we’re truly getting value for money. This would elevate the paper’s relevance and make its contribution to the field more apparent.

We have included sentences which addresses both these points on p3:

As such, we believe that this is among the first studies to provide a detailed financial estimate of treaty-making in global health. This matters as it allows us to open the conversation about whether this is a good use of money, particularly within a context of scarce resources and dwindling political commitment to increase financing for pandemic preparedness and response.

2. The approach to estimating human costs is logical and appropriate, using salary data and time allocations. However, it relies heavily on assumptions, as the authors note. This is necessary based on what information is available. But a sensitivity analysis would help demonstrate the robustness of these estimates. For instance, how might the total cost fluctuate if time allocation assumptions (10%, 25%, 50%) change? Additionally, while I understand there are privacy concerns, providing more transparency about the four-member state delegates consulted for these assumptions would strengthen the credibility of the data.

We have performed a sensitivity analysis on our overall figures to offer the potential for error in the assumptions used for our calculations. We present each of the combined costs with a 5% 10% and 25% margin of error each way, to determine how variability in human hours, salary, event/travel costs may impact our final figures.When taking each margin of error in turn, our final estimate of $201,343,032 for INB sessions 1 to 15 is altered as follows:

±5% equates to ± $10,067,151 leading to upper and lower estimates of $211,410,184 and $191,275,881 respectively.

±10% equates to ± $20,134,303 leading to upper and lower estimates of $221,477,336 and $181,208,729 respectively.

±25% equates to ±$50,335,758 leading to upper and lower estimates of $251,678,791and $151,007,274 respectively.

We note that even in the case of ±25% error margin, the upper bound only greatly exacerbates the severity of the points made below in the discussion, and the lower bound estimate of $151,007,274 is still an incredibly high figure, bearing in mind that this is broadly similar to Japan’s total contributions to WHO of $147 million. Therefore, even in the unlikely case where our total estimate is subject to a 25% error margin, it still merits debate and evaluation on whether this is an efficient allocation of increasingly limited resources for global pandemic preparedness.

We are unable to provide more information about the four member state delegates that we consulted for our assumptions, as this were informal consultations and we do not have their consent to name them/the member states they represent.

3. The method of calculating travel costs, using average flight costs and per diem rates, makes sense and is well thought out. I appreciate the specificity of return flight costs per country. That said, given the volatility of flight prices, this is another area where sensitivity analysis would be beneficial. A robustness check on these assumptions could provide more confidence in the estimates.

In our sensitivity analysis above, the volatility of the costs of return flights have also been included, and their error margin considered in the discussion section.

4. A deeper comparison of the INB process with other international negotiation processes would be valuable. How do the costs of the INB stack up against other similar treaties, not only in financial terms but also in terms of their (promised/achieved) policy impact? This could provide important context and strengthen the overall argument.

This is a good idea – we have added context to compare the promised policy impact of the INB to that of the other two treaties that WHO have governed, the International Health Regulations (2005) and the Framework Convention on Tobacco Control (FCTC). These both have had much promise, but have delivered relatively little in reality, highlighting the limited legal impact of such treaty making in practice. This additional content can be found on p9.

5. The discussion around opportunity costs is one of the strongest parts of the paper but could be developed more. Could this analysis lead to policy recommendations, such as cost thresholds for international negotiations? Could we imagine more cost-efficient models for treaty-making in global health? Or, is treaty-making itself the most effective (or even a worthwhile) tool for governing global health? These questions would deepen the discussion and make the analysis more forward-looking.

We have added to this paragraph raising questions about whether treaties are worthwhile, linking to the increasing evidence eg. Hoffman et al 2022 which demonstrates that treaties have rarely achieved their intended effects. Others, however, consider the value of treaties less in the legalease, and more in the normative interactions that are gained through the process itself, albeit not quantifiable. We hope that this paper in turn sparks a conversation as to value of such a process, and whether detailed budgets not only of WHO’s activities, but the global cost of such a process should be discussed at the start as part of the decision as to whether to proceed through such activities or not. This would then allow for meaningful considerations of opportunity costs, and what potential value the normative and procedural aspects of such negotiations hold.

6. The section on the potential negative impact of the INB process on the WHO and challenges faced by low-income countries is compelling but could be expanded. What recommendations could the authors provide to make future negotiations more equitable and cost-effective? Are there strategies to reduce costs while still achieving meaningful outcomes?

In terms of the WHO, we have added a few sentences to demonstrate the best practice that WHO could have adopted from hundreds of years of multilateral diplomacy in other areas. The fact that WHO departed on its own without embedded tried and tested tactics is in part, one of the reasons WHO has lost credibility through this this process. We propose that potential options could include revolving the hosting of the INB meetings in WHO Regional Offices to share the travel burden, and reduce overall costs given the artificially inflated prices in Geneva.

7. Rather than focusing solely on the financial costs, the conclusion could end with a broader reflection on how these findings can guide future governance processes. How can we ensure resources are efficiently allocated in global health, striking the right balance between governance and operational needs? Ending on this note may help translate the findings into actionable insights.

We have updated the conclusion to make it more reflective in general about what lessons can be learned for governance processes in general.

Recommendation: This paper makes a significant contribution to global health economics by shedding light on the costs of a major international governance process. However, there’s room to strengthen the methodology, clarify assumptions, and expand the policy implications. I recommend major revisions to address these areas.

We hope that our revisions have addressed your concerns and have strengthened the methodology, assumptions and policy implications.
